# Perioperative Blood Glucose Levels <150 mg/dL are Associated With Improved 5-Year Survival in Patients Undergoing On-Pump Cardiac Surgery: A Prospective, Observational Cohort Study: Erratum

**DOI:** 10.1097/01.md.0000481352.86225.10

**Published:** 2016-03-03

In the article “Perioperative Blood Glucose Levels <150 mg/dL are Associated With Improved 5-Year Survival in Patients Undergoing On-Pump Cardiac Surgery: A Prospective, Observational Cohort Study”,^1^ which appeared in Volume 94, Issue 45 of *Medicine*, Dr. Abu Hanna's name was originally presented incorrectly. The article has since been corrected online.
